# Autophagy plays an antiviral defence role against tomato spotted wilt orthotospovirus and is counteracted by viral effector NSs

**DOI:** 10.1111/mpp.70012

**Published:** 2024-09-30

**Authors:** Xingwang Zhang, Hao Hong, Jiaoling Yan, Yulong Yuan, Mingfeng Feng, Qinhai Liu, Yanxiao Zhao, Tongqing Yang, Shen Huang, Chunli Wang, Ruizhen Zhao, Wenyu Zuo, Suyu Liu, Zixuan Ding, Changjun Huang, Zhongkai Zhang, Jiban Kumar Kundu, Xiaorong Tao

**Affiliations:** ^1^ The Key Laboratory of Plant Immunity, Department of Plant Pathology Nanjing Agricultural University Nanjing China; ^2^ Key Laboratory of Tobacco Biotechnological Breeding, National Tobacco Genetic Engineering Research Center Yunnan Academy of Tobacco Agricultural Sciences Kunming China; ^3^ Biotechnology and Germplasm Resources Research Institute, Yunnan Seed Laboratory Yunnan Academy of Agricultural Sciences China; ^4^ Plant Virus and Vector Interactions—Centre for Plant Virus Research, Crop Research Institute Prague Czech Republic; ^5^ Laboratory of Virology—Centre for Plant Virus Research, Institute of Experimental Botany of the Czech Academy of Sciences Prague Czech Republic

**Keywords:** antiviral defence, autophagy, counterdefence, nonstructural protein NSs, nucleocapsid protein, TSWV

## Abstract

Autophagy, an intracellular degradation process, has emerged as a crucial innate immune response against various plant pathogens, including viruses. Tomato spotted wilt orthotospovirus (TSWV) is a highly destructive plant pathogen that infects over 1000 plant species and poses a significant threat to global food security. However, the role of autophagy in defence against the TSWV pathogen, and whether the virus counteracts this defence, remains unknown. In this study, we report that autophagy plays an important role in antiviral defence against TSWV infection; however, this autophagy‐mediated defence is counteracted by the viral effector NSs. Transcriptome profiling revealed the up‐regulation of autophagy‐related genes (ATGs) upon TSWV infection. Blocking autophagy induction by chemical treatment or knockout/down of *ATG5*/*ATG7* significantly enhanced TSWV accumulation. Notably, the TSWV nucleocapsid (N) protein, a major component of the viral replication unit, strongly induced autophagy. However, the TSWV nonstructural protein NSs was able to effectively suppress N‐induced autophagy in a dose‐dependent manner. Further investigation revealed that NSs inhibited ATG6‐mediated autophagy induction. These findings provide new insights into the defence role of autophagy against TSWV, a representative segmented negative‐strand RNA virus, as well as the tospoviral pathogen counterdefence mechanism.

## Graphical abstract

Autophagy plays an important role in antiviral defence against tomato spotted wilt orthotospovirus (TSWV) infection. However, the TSWV NSs is able to effectively suppress N‐induced autophagy in a dose‐dependent manner.

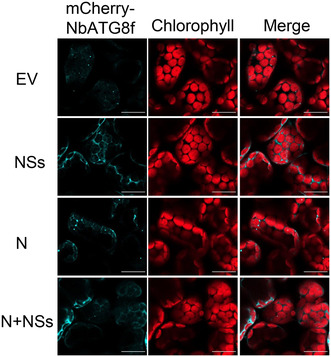

## 1 INTRODUCTION

Plants have evolved different defence mechanisms to combat plant pathogens, including viruses. Two major mechanisms involved in host innate immunity against virus infections are RNA interference (RNAi), also known as RNA silencing, and resistance (R) gene‐mediated effector‐triggered immunity (ETI) in eukaryotes (Mandadi & Scholthof, 2013). RNA silencing provides a basal antiviral immunity to plant viruses by allowing the host RNA silencing machinery to recognize viral double‐stranded RNA (dsRNA) and defend host cells in a sequence‐specific manner. Viral dsRNA serves as a typical pathogen‐associated molecular pattern (PAMP), making RNA silencing akin to pattern‐triggered immunity (PTI) (Ding, 2010). However, viruses have developed viral suppressor proteins to counteract the host RNA silencing and facilitate their own infection. In response, plants have also evolved intracellular immune receptors to trigger a more robust defence response (ETI) by recognizing viral effector proteins. ETI often leads to a hypersensitive response (HR), resulting in localized cell death that restricts the spread of viruses (Meier et al., 2019).

Autophagy is an evolutionarily conserved intracellular process that occurs during stress conditions or specific developmental processes. During autophagy, double membrane‐bound autophagosome vesicles are formed to enclose and transport cytoplasmic content to the lysosome/vacuole for degradation and recycling (Mizushima et al., 2008; Ohsumi, 2001; Qi et al., 2021). Through extensive genetic screens of yeast mutants, about 40 autophagy‐related genes (ATGs) have been identified (Boya et al., 2013; Kellner et al., 2017; Wen & Klionsky, 2016). The formation of autophagosomes relies on a core set of autophagy‐related proteins (Mizushima & Komatsu, 2011). Recent research has shown that autophagy also contributes to plant innate immunity against plant viruses, including geminiviruses (Haxim et al., 2017; Li et al., 2020), cauliflower mosaic virus (CaMV) (Hafrén et al., 2017; Hafrén & Hofius, 2017), turnip mosaic virus (TuMV) (Hafrén et al., 2018), and rice stripe virus (RSV) (Jiang et al., 2021; Zhao et al., 2023). Selective autophagy has been found to limit CaMV and geminivirus infection through NBR1‐mediated degradation of coat protein (CP) and virion particles of CaMV or βC1 encoded by geminiviruses (Hafrén et al., 2017; Zhou et al., 2021). However, some plant viruses have evolved mechanisms to counteract antiviral autophagy (Hafrén et al., 2018; Yang et al., 2018). For example, TuMV counteracts selective autophagy using Hc‐Pro, a viral suppressor of RNA silencing. The γb protein of barley stripe mosaic virus (BSMV) disrupts vacuolar acidification and subverts autophagy to facilitate virus infection in plants (Yang et al., 2018).

Tomato spotted wilt orthotospovirus (TSWV) is a representative segmented negative‐strand RNA plant virus that belongs to the family *Tospoviridae* in the order *Bunyavirales* (Adams et al., 2017). TSWV is a member of the genus *Orthotospovirus*, which currently comprises more than 30 species (Kormelink et al., 2021). TSWV is the most extensively studied virus in this family and has a wide host range, infecting over 1000 plant species worldwide (Kormelink et al., 2011). Its ability to infect numerous plant species makes TSWV one of the most destructive plant viruses, posing a significant threat to global food production (Scholthof et al., 2011). The TSWV genome is composed of three segments: large (L), medium (M), and small (S) RNA segments. The L RNA segment is negatively polar and encodes the RNA‐dependent RNA polymerase (RdRp) (Adkins et al., 1995). Both the M and S segments use an ambisense strategy to encode viral proteins. The viral (v) strand of the M RNA segment encodes a nonstructural protein (NSm), which plays a crucial role in cell‐to‐cell and long‐distance movement within plants (Feng et al., 2016; Kormelink et al., 1994; Qian et al., 2018; Soellick et al., 2000; Storms et al., 1995, 1998). The viral complementary (vc) strand of the M segment encodes a precursor for glycoproteins, which are further processed into two mature glycoproteins, Gn and Gc. These glycoproteins are presented on the surface of enveloped virus particles and are essential for virus transmission from infected plants to healthy plants via thrips vectors (Kikkert et al., 1999; Sin et al., 2005). The v strand of the S RNA segment encodes a nonstructural protein (NSs), which acts as an RNA silencing suppressor and plays a critical role in counteracting plant immune responses mediated by RNAi (Bucher et al., 2003; Schnettler et al., 2010; Takeda et al., 2002). The vc strand of the S segment encodes the nucleocapsid protein (N), which assembles the genomic RNAs into ribonucleoproteins (RNPs) (Feng et al., 2016; Guo et al., 2017; Komoda et al., 2017). RNPs associate with RdRp and represent the minimal functional units for viral replication (Feng et al., 2020; Walter et al., 2011).

While both RNAi and ETI have been shown to be crucial for antiviral defences against TSWV, the role of autophagy in defence against TSWV and whether any viral encoded proteins counteract this defence remains unknown. In this study, we demonstrate that autophagy plays an important role in antiviral defence against TSWV infection. However, this defence is suppressed by the NSs protein encoded by TSWV.

## 2 RESULTS

### 2.1 TSWV infection activates autophagy in host plants

**FIGURE 1:**
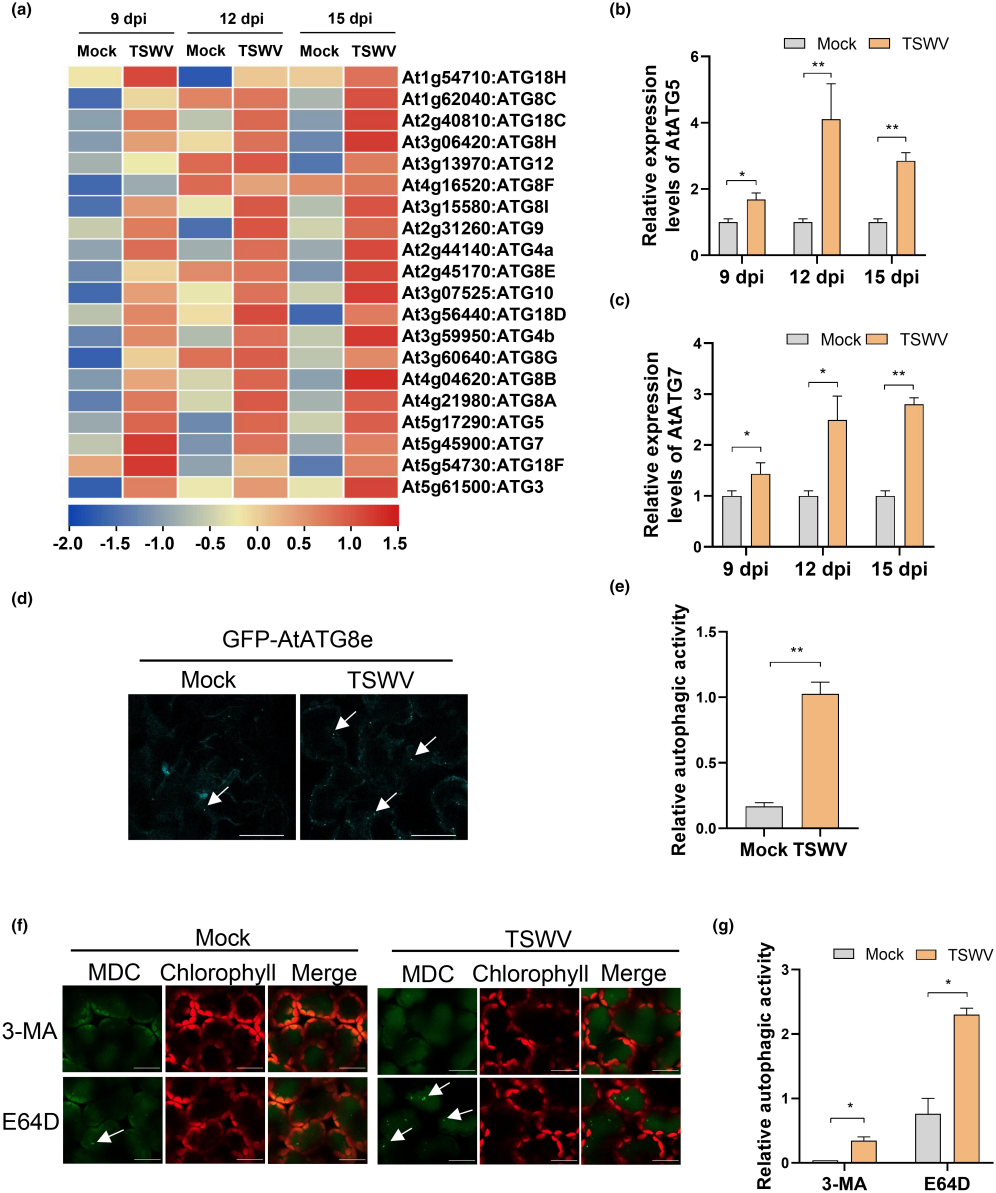
TSWV infection activates autophagy in host plant. (a) Heatmap illustrating the expression of genes involved in autophagy pathways analysed from RNA‐seq data of mock‐inoculated and TSWV‐infected *Arabidopsis thaliana* plants 9, 12 and 15 days post‐inoculation (dpi). (b) and (c) Relative expression of *AtATG5* and *AtATG7* in mock‐inoculated or TSWV‐infected *A. thaliana* as determined by reverse transcription‐quantitative PCR at 9, 12 and 15 days dpi. The results are represented as the means (±*SD*) of three replicates. *p < 0.05, **p < 0.01, Student's *t* test. (d) Detection of autophagosomes in the GFP‐AtATG8e transgenic *A. thaliana* line induced by TSWV at 12 dpi. Mock inoculation was used as a control. Bars, 20 μm. (e) and (g) Relative autophagy activity revealed by the GFP‐AtATG8e transgenic *A. thaliana* line (d) and MDC‐staining (f) in TSWV‐infected leaves was normalized to that of mock‐inoculated leaves. Quantification of GFP‐AtATG8e‐ or monodansylcadaverine (MDC)‐labelled autophagic puncta per cell was performed by counting the autophagic bodies. More than 100 cells per treatment were used for quantification. *p < 0.05, **p < 0.01, Student's *t* test. (f) Relative autophagic puncta labelled by MDC‐staining in mock‐ or TSWV‐infected leaves treated with 50 μM E‐64d or 5 mM 3‐MA. Bars, 20 μm. Error bars indicate *SE* from three independent samples.

To investigate whether autophagy is induced during TSWV infection, we analysed the previously characterized high‐throughput transcription profiles of *Arabidopsis thaliana* infected with TSWV at 9, 12, and 15 days post‐inoculation (dpi) (Xu et al., 2020). The results showed that the expression levels of numerous ATGs were upregulated in TSWV‐infected *Arabidopsis* at 9, 12, and 15 dpi (Figure 1a). To further validate these observations, we conducted reverse transcription‐quantitative PCR (RT‐qPCR) analysis to examine the expression of two core autophagy genes, *AtATG5* and *AtATG7*, in newly emerged leaves of TSWV‐infected *A. thaliana* at 9, 12, and 15 dpi. The results confirmed a significant upregulation of *AtATG5* and *AtATG7* expression in TSWV‐infected new leaves at 12 and 15 dpi, although no significant upregulation was observed at 9 dpi (Figure 1b,c).

To directly observe autophagy induction during TSWV infection, we used a transgenic *Arabidopsis* line expressing an autophagosome marker GFP‐AtATG8e (Contento et al., 2005). As depicted in Figure 1d,e, the number of punctate GFP‐ATG8 fluorescent structures in TSWV‐infected systemic leaves at 12 dpi was more than six‐fold higher compared to the mock‐inoculated plants (Figure 1d,e). To further validate these observations, we assessed autophagy induction activity in TSWV‐infected *Nicotiana benthamiana* systemic leaves through monodansylcadaverine (MDC) staining after treatment with E‐64d, a cysteine protease inhibitor that prevents autophagic vacuolar degradation. MDC staining revealed a prominent increase in autophagic activation in TSWV‐infected leaves compared to mock‐inoculated controls. In contrast, leaves treated with 3‐MA, an autophagy‐specific inhibitor, showed no autophagosome structures during TSWV‐infection at 12 dpi (Figure 1f,g).

### 2.2 Autophagy plays a critical antiviral role during TSWV infection

**FIGURE 2:**
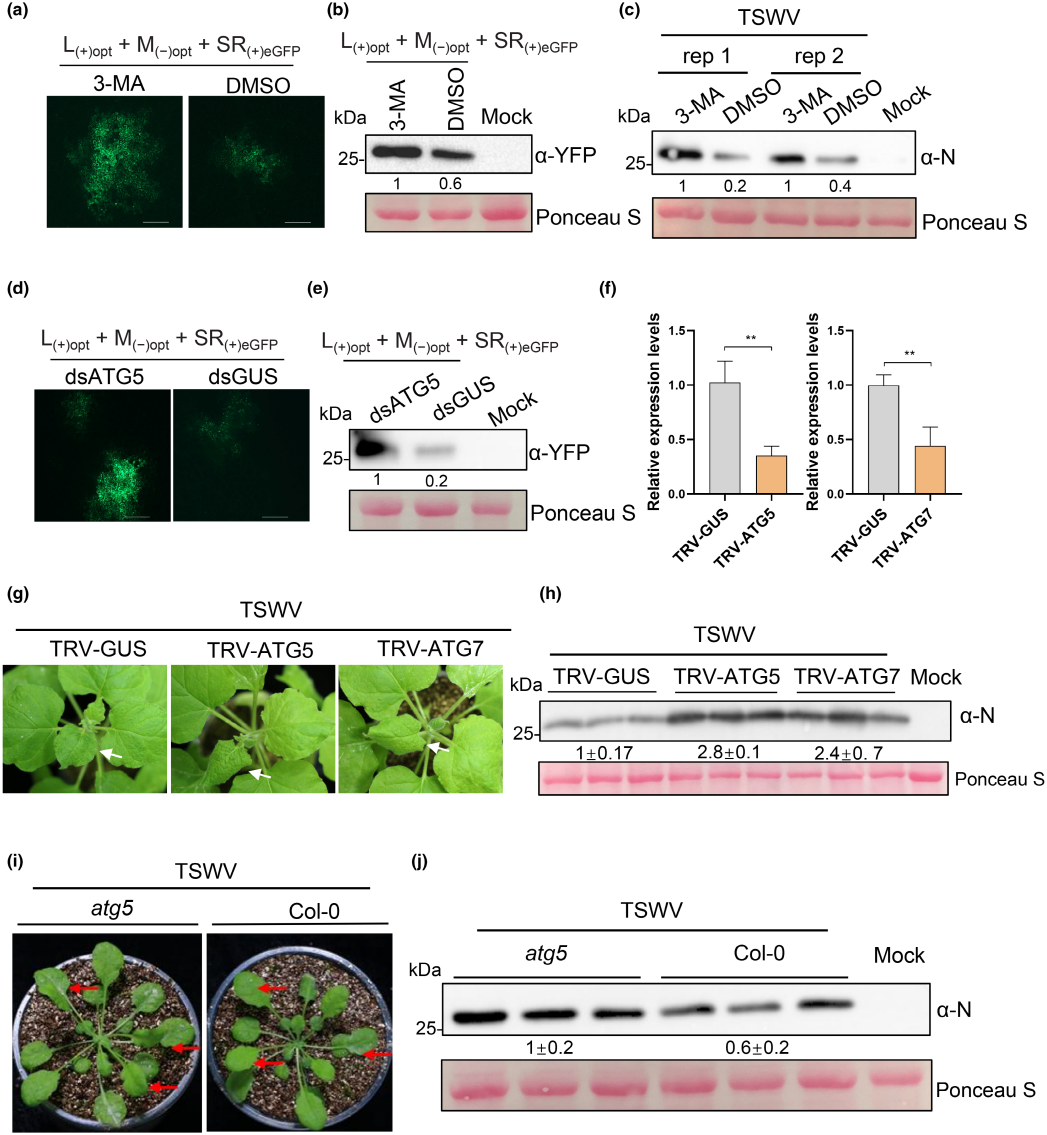
Autophagy plays an antiviral defence role against TSWV infection. (a) 3‐MA treatment enhances TSWV infection in *Nicotiana benthamiana* plant. *N. benthamiana* plant leaves were treated with 3‐MA for 12 h and followed with agro‐inoculation of TSWV infectious clones L_(+)opt_ + M_(−)opt_ + SR_(+)eGFP_. eGFP fluorescence from TSWV were examined at 48 hpi under an inverted fluorescence microscope. Bars, 200 μm. (b) Western blot showing the accumulation level of TSWV in (a) using an anti‐GFP antibody. (c) *N. benthamiana* leaves treated with 3‐MA were also inoculated with sap of fresh TSWV‐infected tissue. Western blot results showing the accumulation level of N in TSWV‐inoculated leaves using a N‐specific antibody. (d) Silencing *NbATG5* by hairpin RNA construct (RNAi‐*NbATG5*) enhanced TSWV accumulation in *N. benthamiana*. *N. benthamiana* leaves co‐expressing dsATG5 or dsGUS hairpin RNA constructs with TSWV infectious clones L_(+)opt_ + M_(−)opt_ + SR_(+)eGFP_ in *N. benthamiana* leaves. eGFP fluorescence from TSWV infectious clones were examined at 60 h post‐inoculation under an inverted fluorescence microscope. Bars, 200 μm. (e) Western blot showing the accumulation level of TSWV in (d) using an anti‐GFP antibody. (f) Relative expression levels of *NbATG5* or *NbATG7* in *N. benthamiana* plants pretreated with TRV‐*NbATG5* and TRV‐*NbATG7*, respectively, by reverse transcription‐quantitative PCR (means ± *SD*, *n* = 3). (g) Phenotypes of the *NbATG5*‐ and *NbATG7*‐silenced plants infected with TSWV at 6 days post‐inoculation (dpi). (h) Accumulation of TSWV in *NbATG5‐* or *NbATG7*‐silenced plants analysed by western blot using N‐specific antibodies at 6 dpi. (i) Phenotypes of the *atg5* mutant and wild‐type (WT) *Arabidopsis thaliana* infected with TSWV at 12 dpi. (j) The accumulation of TSWV N protein in *atg5* and WT plants analysed by western blot at 12 dpi.

To explore the role of autophagy in TSWV infection, we treated *N. benthamiana* plants with the autophagy inhibitor 3‐MA and subsequently agro‐inoculated them with infectious clones of TSWV L_(+)opt_ +M_(−)opt_ +SR_(+)eGFP_ carrying a green fluorescent protein (GFP) reporter, which we developed previously (Feng et al., 2020). The results showed a significant increase in GFP expression from TSWV in 3‐MA‐treated leaves compared to dimethyl sulphoxide (DMSO)‐treated control leaves (Figure 2a,b, Figure S1). Furthermore, we inoculated the 3‐MA‐treated *N. benthamiana* plants with sap from TSWV‐infected tissue. Immunoblot analysis revealed a marked elevation in TSWV accumulation in 3‐MA‐treated leaves compared to control leaves (Figure 2c). To investigate the role of autophagy during TSWV infection, we expressed a hairpin RNA construct to silence *NbATG5* (RNAi‐*NbATG5*) in *N. benthamiana* plants followed by inoculation with infectious clones of TSWV L_(+)opt_ +M_(−)opt_ +SR_(+)eGFP_ (Figure S1). A hairpin RNA construct targeting *β‐glucuronidase* (*GUS*) (RNAi‐*GUS*) was used as a control. As shown in Figure 2d,e, the accumulation of TSWV was significantly higher in leaves treated with RNAi‐*NbATG5* compared to control leaves (Figure 2d,e).

To further examine the role of autophagy during TSWV infection, we silenced two core autophagy machineries, *ATG5* and *ATG7*, in *N. benthamiana* plants through tobacco rattle virus (TRV)‐induced gene silencing (Figure 2f). At 14 days after TRV treatment, we inoculated the plants with sap of TSWV. At 6 days after TSWV inoculation, the *ATG5*‐ and *ATG7*‐silenced plants exhibited more severe viral symptoms in newly emerged leaves compared to TRV‐*GUS*‐treated control plants (Figure 2g). Immunoblot and RT‐qPCR analysis confirmed a significant increase in TSWV accumulation in *ATG5*‐ and *ATG7*‐silenced plants compared to TRV‐*GUS*‐treated control plants (Figure 2h, Figure S2). To further validate these findings, we inoculated TSWV onto wild‐type (WT) *A. thaliana* plants and *atg5* knockout mutant. At 12 dpi, more severe disease symptoms were observed in the *atg5* knockout mutants compared to the WT plants (Figure 2i). Immunoblot results further confirmed that TSWV accumulation was significantly higher in the *atg5* mutant compared to that in WT plants (Figure 2j). Collectively, these findings suggest that autophagy plays a crucial role in antiviral defence against TSWV infection.

### 2.3 TSWV nucleocapsid protein strongly induces autophagy

**FIGURE 3:**
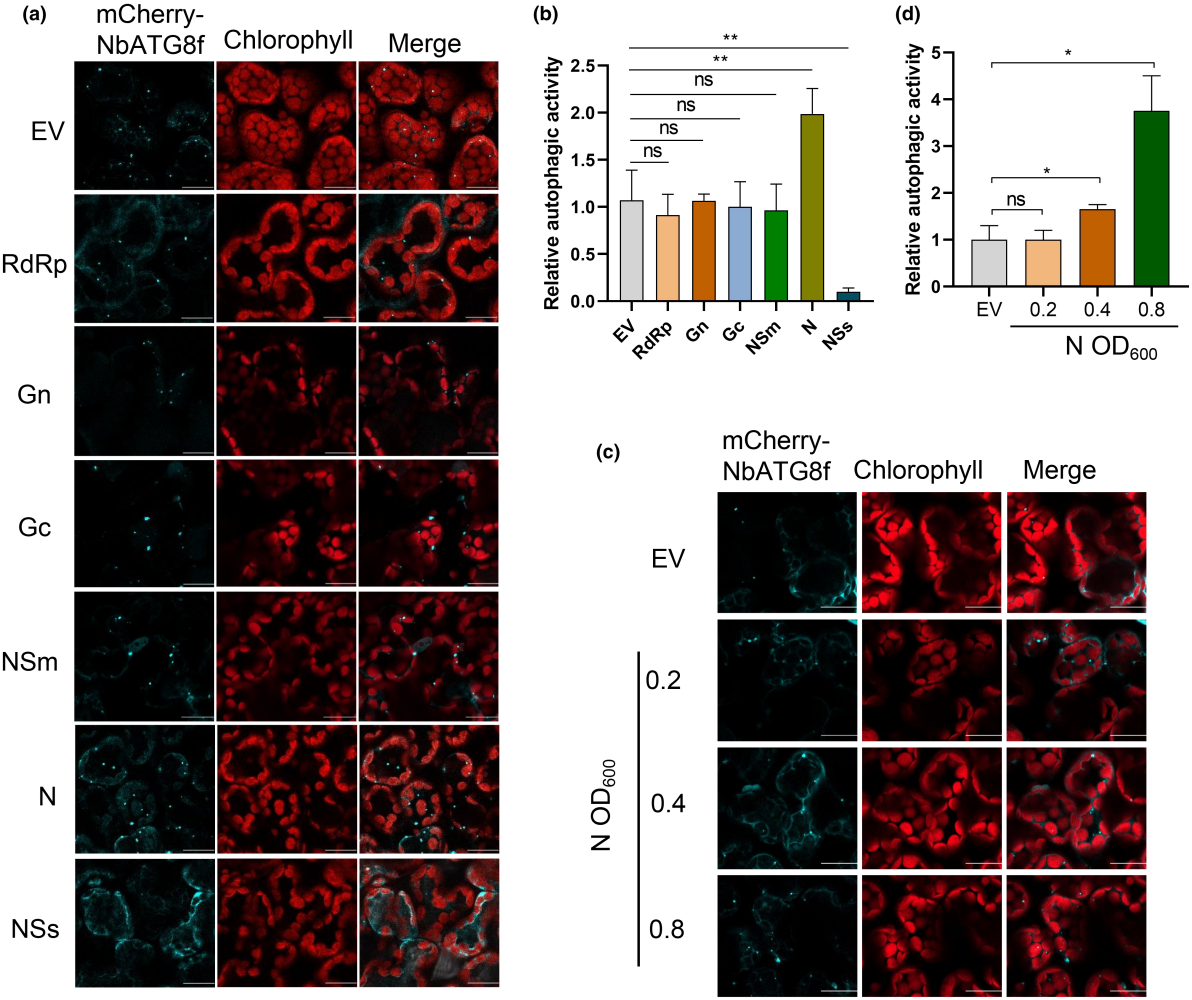
TSWV N strongly induces autophagy, whereas TSWV NSs suppresses the induction of autophagy. (a) Induction analysis of mCherry‐NbATG8f‐labelled autophagic puncta by expression of TSWV RdRp, Gn, Gc, NSm, N, NSs or a pCambia2300 empty vector (EV) control in *Nicotiana benthamiana*. Bars, 20 μm. (b) Relative autophagy activity by TSWV RdRp, Gn, Gc, NSm, N and NSs in (a) was normalized to that of leaves expressing the EV control. Quantification of mCherry‐NbATG8f‐labelled autophagic puncta per cell was performed by counting the autophagic bodies. More than 100 cells per treatment were used for quantification. ns, not significant; **p < 0.01, Student's *t* test. (c) Inhibition analysis of autophagy by increasing amount of N. The concentration of *Agrobacterium* carrying N was OD_600_ 0.2, 0.4 or 0.8. mCherry‐NbATG8f was used to label the autophagosome. (d) Quantification of mCherry‐NbATG8f‐labelled autophagic puncta in (c). More than 100 cells per treatment were used for quantification. ns, not significant; *p < 0.05, Student's *t* test.

Having established that TSWV infection triggers autophagy, our next aim was to determine which viral protein encoded by TSWV is responsible for inducing autophagy in the host. We co‐expressed TSWV RdRp, Gn, Gc, NSm, N, NSs, and pCambia2300 empty vector (EV) control with an autophagy‐specific marker, mCherry‐ATG8f, in *N. benthamiana* plants treated with E‐64d. Interestingly, only TSWV N strongly activated autophagy, while the other viral proteins had no significant effect on autophagy induction (Figure 3a,b). RdRp, Gn and Gc were not detected due to either their low expression levels or the poor quality of the corresponding antibodies, but N, NSs and NSm proteins were easily detected (Figure S3). Strikingly, TSWV NSs was found to strongly suppress the induction of autophagy (Figure 3a,b). These results suggest that TSWV N can induce autophagy activity, while viral NSs actively suppresses the induction of autophagy. To evaluate whether N has dose‐dependent inhibition activity on autophagy, we adjusted the concentration of agrobacterium carrying N to OD_600_ 0.1, 0.2, 0.4, and 0.8. As shown in Figure 3c,d, the concentrations of N at OD_600_ 0.2 did not induce autophagy. However, when the concentration of N increased with bacterial concentrations from OD_600_ 0.4 to 0.8, a distinct induction of the punctate structures was observed (Figure 3c,d). These results suggest a dose‐dependent induction effect of N on autophagy.

### 2.4 TSWV NSs inhibits N‐induced autophagy

**FIGURE 4:**
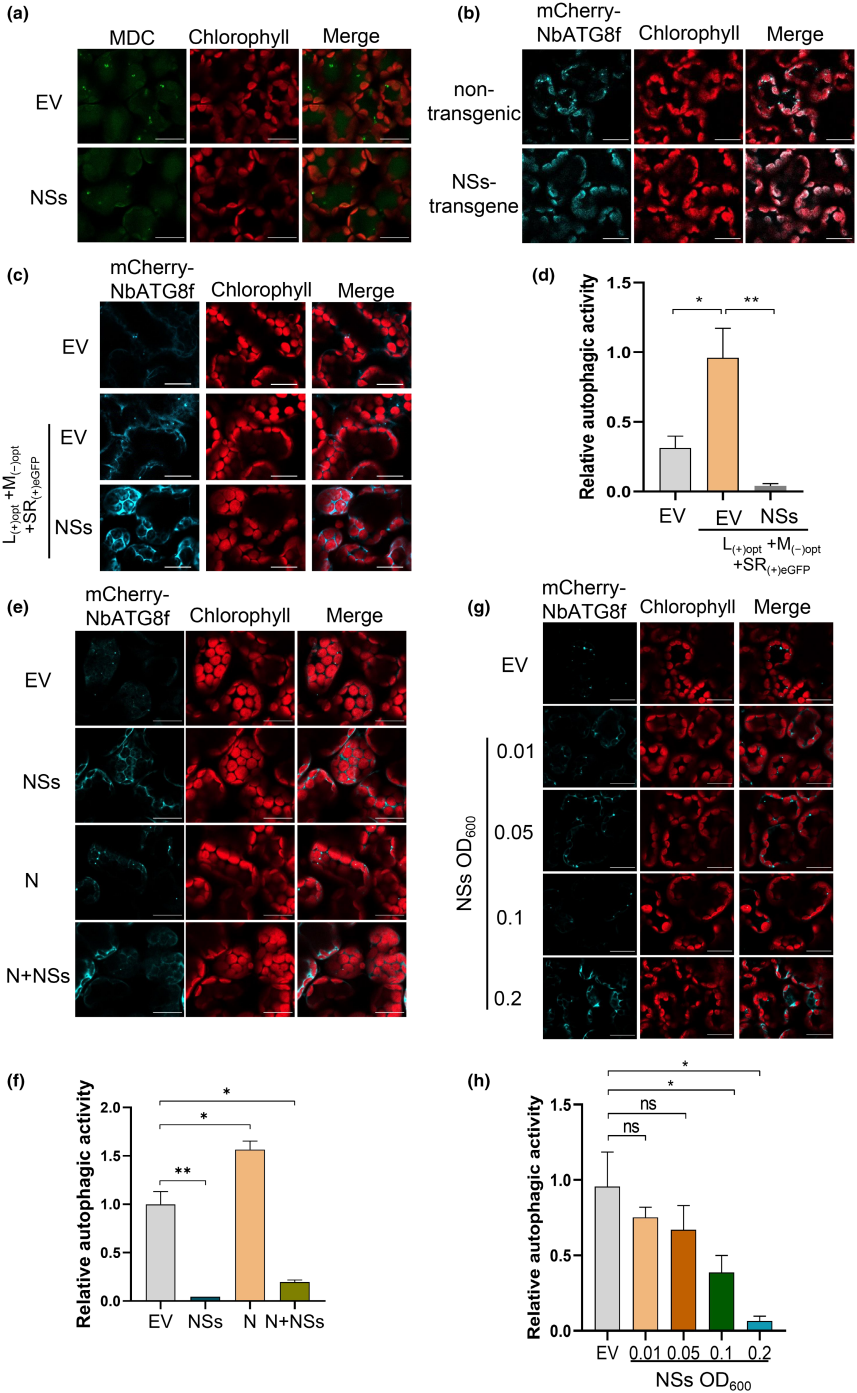
TSWV NSs inhibits N‐induced autophagy. (a) Representative images of autophagic puncta stained by monodansylcadaverine (MDC) in cells transiently expressing NSs or empty vector (EV). Bars, 20 μm. (b) Representative images of mCherry‐NbATG8f‐labelled autophagosome in NSs‐transgenic and non‐transgenic *Nicotiana benthamiana* plants. Bars, 20 μm. The autophagic marker mCherry‐NbATG8f was transiently expressed in these *N. benthamiana* leaves. (c) Relative autophagic activity of TSWV L_(+)opt_ + M_(−)opt_ + SR_(+)eGFP_ in which NSs were replaced with GFP in *N. benthamiana* leaves in the absence (EV) or the presence of NSs. mCherry‐NbATG8f was used to label the autophagic puncta. bars, 20 μm. (d) Relative autophagy activity revealed by mCherry‐NbATG8f was normalized to that of EV or mock‐inoculated leaves shown in panel (c). Quantification of mCherry‐NbATG8f autophagic puncta per cell was performed by counting the autophagic bodies. More than 100 cells per treatment were used for quantification. *p < 0.05, **p < 0.01, Student's *t* test. (e) Co‐expression of the mCherry‐ATG8f with EV, NSs, N and N + NSs in *N. benthamiana* leaves via agroinfiltration. Bars, 20 μm. (f) Quantification of mCherry‐NbATG8f‐labelled autophagic puncta in (a). More than 100 cells per treatment were used for quantification. *p < 0.05, **p < 0.01, Student's *t* test. (g) Inhibition analysis of autophagy by increasing the amount of NSs. The concentration of *Agrobacterium* carrying NSs was OD_600_ 0.01, 0.05, 0.1 or 0.2. mCherry‐NbATG8f was used to label the autophagosome. (h) Quantification of mCherry‐NbATG8f‐labelled autophagic puncta in (c). More than 100 cells per treatment were used for quantification. ns, not significant; *p < 0.05, Student's *t* test.

Next, we stained the autophagic granules using MDC, and similar inhibition of autophagy was observed in the presence of NSs (Figure 4a). To further confirm these findings, we generated transgenic *N. benthamiana* plants that stably expressed NSs. We expressed the autophagy‐specific marker mCherry‐NbATG8f in the leaves of these transgenic plants followed with treatment of E‐64d. The results showed a significant reduction in autophagic granules in NSs‐expressing leaf tissues compared to leaves expressing the EV control vector (Figure 4b).

To investigate whether TSWV infection without NSs could induce autophagy and whether NSs counteracts it, we expressed TSWV L_(+)opt_ +M_(−)opt_ +SR_(+)eGFP_, in which NSs was replaced with eGFP, in *N. benthamiana* leaves (Figure S1). As shown in Figure 4c,d, leaf tissues infected with TSWV L_(+)opt_ +M_(−)opt_ +SR_(+)eGFP_ lacking NSs exhibited a significant increase in autophagosome induction compared to control leaves, while the accumulation of autophagic bodies was largely inhibited in the presence of NSs (Figure 4c,d).

As TSWV N can induce autophagy, we then examine whether TSWV NSs has an inhibitory effect on N‐induced autophagy. mCherry‐*Nb*ATG8f was co‐expressed with different constructs, including EV, NSs, N, and N + NSs in *N. benthamiana* leaves. Fluorescence microscopy analysis revealed that the expression of N alone led to a significant increase in punctate fluorescent structures labelled by mCherry‐NbATG8 compared to leaves expressing EV. In contrast, co‐expression of N and NSs resulted in a significant reduction in the number of autophagic punctate structures, indicating that NSs can interfere with the induction of autophagy mediated by N (Figure 4e,f).

To evaluate whether NSs has dose‐dependent inhibition activity on autophagy, we adjusted the concentration of *Agrobacterium* carrying NSs to OD_600_ 0.01, 0.05, 0.1, or 0.2. As shown in Figure 4c,d, the concentrations of NSs at OD_600_ 0.01 and 0.05 did not inhibit autophagy. However, when the concentration of NSs increased from OD_600_ 0.1 to 0.2, a complete inhibition of the punctate structures was observed (Figure 4g,h). These results suggest a dose‐dependent inhibitory effect of NSs on autophagy.

### 2.5 The efficiency of NSs in inhibiting autophagy is dependent on the accumulation of N

**FIGURE 5:**
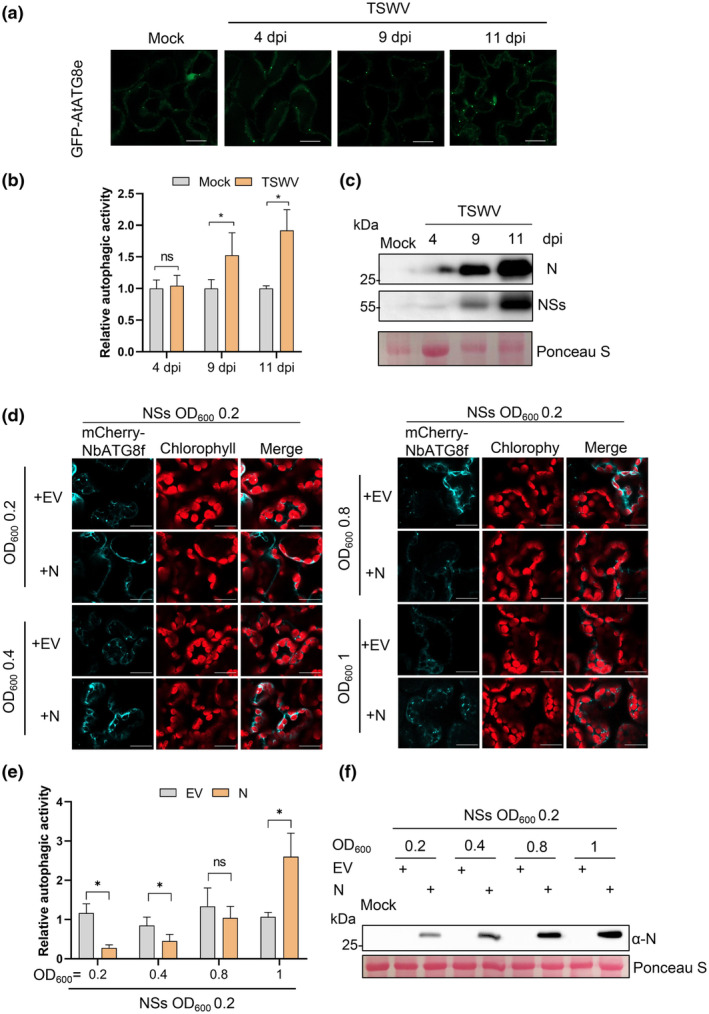
The efficiency of NSs in inhibiting autophagy depends on the accumulation of N. (a) Autophagic activity induced by TSWV in GFP‐AtATG8e transgenic *Arabidopsis thaliana* line at 4, 9 or 11 days post‐inoculation (dpi). Mock inoculation was used as a control. Bars, 20 μm. (b) Quantification of GFP‐AtATG8e‐labelled autophagic puncta in (c). More than 100 cells per treatment were used for quantification. ns, not significant; *p < 0.05, Student's *t* test. (c) Western blot results showing the accumulation level of N or NSs in the infiltrated leaves. (d) *Agrobacterium* carrying N was fixed at OD_600_ of 0.2 and *Agrobacterium* carrying NSs was co‐expressed at OD_600_ 0.2, 0.4, 0.8 and 1.0. Relative autophagy activity of different combinations of N and NSs revealed by mCherry‐NbATG8f was normalized to that of leaves expressing empty vector (EV). (e) Quantification of mCherry‐NbATG8f‐labelled autophagic puncta per cell was performed by counting the autophagic bodies. More than 100 cells per treatment were used for quantification. ns, not significant; *p < 0.05, Student's *t* test. (f) Western blot results showing the accumulation level of N in the infiltrated leaves shown in (a) using N‐specific antibody at 60 h post‐inoculation.

To investigate whether autophagy induction is concomitant with increased N accumulation during the late stage of infection, we inoculated the GFP‐AtATG8e transgenic *A. thaliana* line with sap from TSWV‐infected tissue. We analysed the accumulation of punctate GFP‐AtATG8 fluorescent structures at 4, 9 and 11 dpi to compare autophagy levels at different infection stages. The results showed no significant difference in autophagy induction levels between TSWV‐infected and mock control samples at 4 dpi (Figure 5a,b). However, autophagy levels were increased in TSWV‐infected leaves at 9 and 11 dpi compared to mock‐inoculated leaves (Figure 5a,b). Western blot showed that accumulation of both TSWV N and NSs increased during the onset of virus infection (Figure 5c).

The ability of NSs to suppress autophagy depends on its accumulation levels, as depicted in Figure 3c,d. To investigate the relationship between the efficacy of NSs in inhibiting autophagy and the relative accumulation of N, we co‐expressed NSs with increasing concentrations of N. *Agrobacterium* carrying NSs was maintained at a fixed OD_600_ 0.2, while *Agrobacterium* carrying N was co‐expressed at OD_600_ values 0.2, 0.4, 0.8, or 1.0. The results showed that NSs at OD_600_ 0.2 was able to reduce punctate autophagic structures induced by N at OD_600_ 0.2 and 0.4 (Figure 5d–f). However, with an increase in N concentration from OD_600_ 0.8 to 1.0, a significant increase in punctate autophagic structures induced by N was observed, indicating that NSs at an OD_600_ of 0.2 was no longer effective in inhibiting autophagy (Figure 5d–f). The relative accumulation levels of N and NSs were also determined at different time points after TSWV inoculation. The results showed that during the natural infection process of TSWV, the ratio of N to NSs showed a gradually increasing trend (Figure S4). These findings suggest that the efficiency of NSs in suppressing autophagy is contingent on the relative accumulation of N.

### 2.6 NSs suppresses the induction of autophagy mediated by ATG6

**FIGURE 6:**
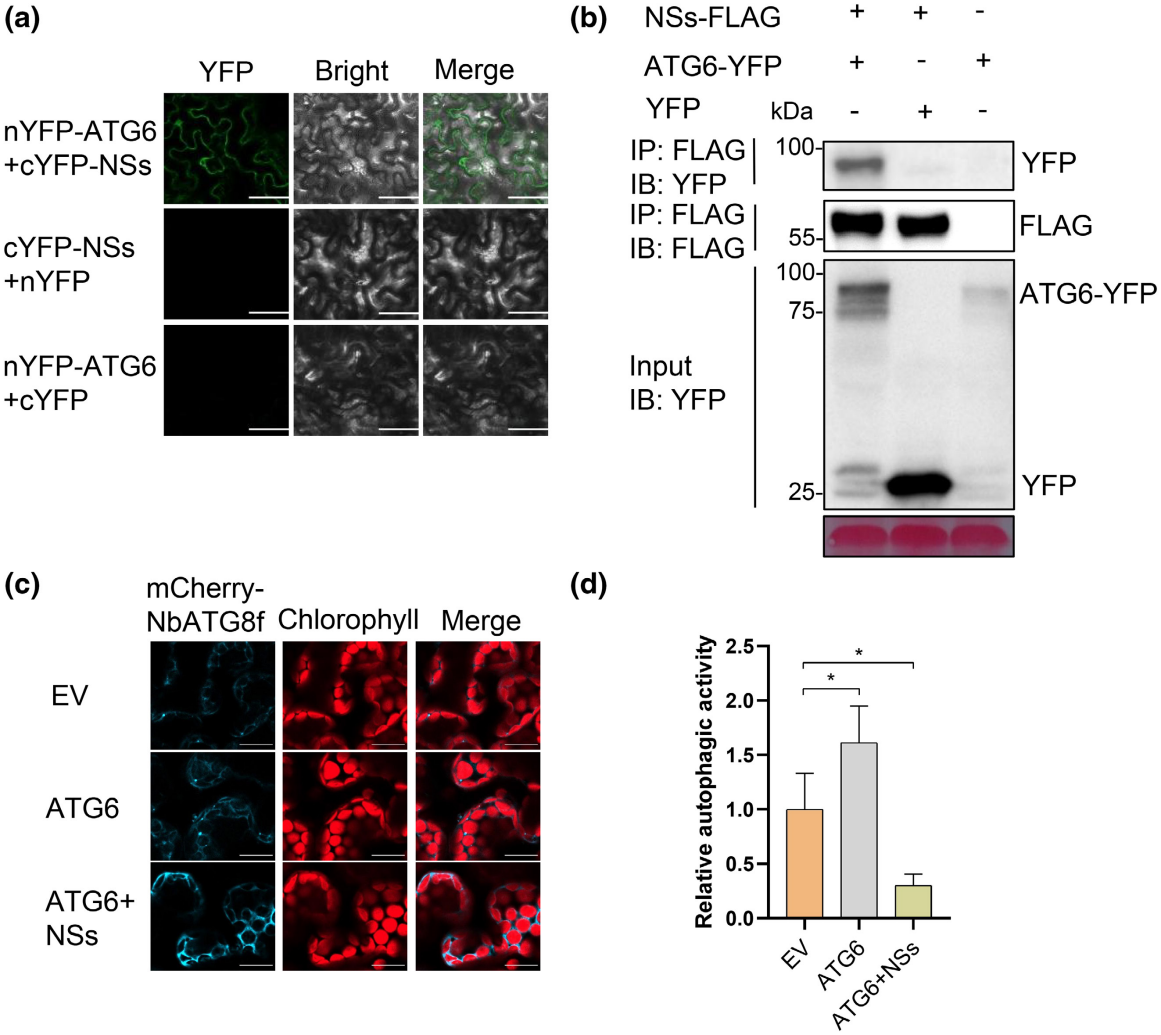
NSs inhibits autophagy induction mediated by ATG6. (a) Bimolecular fluorescence complementation assay for the interaction between ATG6 and NSs in *Nicotiana benthamiana* at 48 h post‐inoculation. Bar, 50 μm. (b) Co‐immunoprecipitation assay for the interaction between NSs and ATG6. FLAG‐tagged NSs was used to immunoprecipitate ATG6‐YFP that was transiently expressed in *N. benthamiana* leaves. The blot was probed using anti‐FLAG_ antibodies. (c) Representative confocal images of autophagic activity in *N. benthamiana* leaf cells (co)expressing autophagy marker mCherry‐NbATG8f with empty vector (EV), ATG6 or ATG6 and NSs. EV‐agroinfiltrated leaves served as the negative control. Bars, 20 μm. (d) Quantification of mCherry‐NbATG8f‐labelled autophagic puncta per cell in (c). More than 100 cells per treatment were used for quantification. *p < 0.05, Student's *t* test.

It has been reported that BSMV γb protein suppresses autophagy by disrupting the ATG7–ATG8 interaction (Yang et al., 2018) and Chinese wheat mosaic virus (CWMV) CP19K inhibits autophagy through enhancing the interaction between GAPC and ATG3 (Niu et al., 2022). To characterize the mechanism by which NSs suppresses autophagy, we performed yeast two‐hybrid (Y2H) assays between NSs and core ATG proteins. However, no direct interactions between NSs and core ATGs were found (Figure S5). Next, we performed a bimolecular fluorescence complementation (BiFC) assay for the interactions between these core ATG proteins and NSs. The results showed that NSs interacted with ATG6, ATG8 and other ATG‐related proteins in planta (Figure S6). We then examined whether NSs can disrupt the ATG7–ATG8 interaction or enhance the GAPC–ATG3 interaction. Neither an inhibition effect on the ATG7–ATG8 interaction nor an enhancing effect on the GAPC1–ATG3 interaction was observed for NSs (Figure S7). We have also investigated the NSs effect on the interactions among other ATG‐related proteins. No significant effect was detected in these ATG interactions (Figure S7). In addition to BiFC (Figure 6a), co‐immunoprecipitation (Co‐IP) analysis also showed that ATG6 interacted with NSs in planta (Figure 6b). A previous study has shown that overexpression of ATG6 activates autophagy in plants (Li et al., 2018). Similarly, we found that the overexpression of ATG6 also induced autophagy compared to the EV control in *N. benthamiana* leaves. However, the number of ATG6‐induced autophagic bodies was significantly reduced in the presence of NSs (Figure 6c,d), suggesting that NSs interferes with autophagy induction mediated by ATG6.

## 3 DISCUSSION

In this study, we demonstrate that autophagy plays an important role in the antiviral defence against TSWV. However, this defence is counteracted by the orthotospoviral pathogen effector NSs. TSWV infection induces the up‐regulation of a large number of ATGs and the induction of autophagosomes in host plants. Knockout/down of *ATG5* or *ATG7* enhanced TSWV infection in host plants. We also found that among the viral proteins encoded by TSWV, the N protein induced autophagy. However, TSWV NSs strongly inhibited N‐induced autophagy and the efficiency of NSs in inhibiting autophagy was dose‐dependent.

TSWV is one of the most important negative‐strand RNA viruses in plants. Silencing of *ATG5* or *ATG7* significantly increased the accumulation and disease symptoms of TSWV in *N. benthamiana* plants, and a similar effect was observed in the *atg5* mutant in *A. thaliana*. Silencing of *ATG5/7* genes has been shown to enhance viral accumulation and disease symptoms in plants infected with single‐stranded DNA viruses including cotton leaf curl Multan virus (CLCuMuV), tomato yellow leaf curl virus (TYLCV), tomato yellow leaf curl China virus (TYLCCNV), and the double‐stranded DNA virus CaMV (Hafrén et al., 2017; Haxim et al., 2017; Ismayil et al., 2020). Similarly, suppression of *ATG5/7* genes increases viral accumulation and disease symptoms in plants infected with positive‐strand RNA viruses such as BSMV and TuMV (Li et al., 2018; Yang et al., 2018). These data highlight the important role of autophagy in the antiviral defence against various types of plant viruses, including single‐stranded and double‐stranded DNA viruses, positive‐strand RNA viruses, and negative‐sense RNA viruses. Previous studies have also shown that autophagy plays a role in defence against RSV (Zhang et al., 2021), another segmented negative‐strand RNA virus. These findings suggest that autophagy may confer broad‐spectrum resistance to different plant negative‐strand RNA viruses.

For negative‐strand RNA viruses, the RNP complex is the minimal replication unit that is assembled by wrapping viral genomic RNA with N protein and it plays an essential role in viral replication and transcription (Hong et al., 2021). N protein is the major component of the viral RNP (Guo et al., 2017; Komoda et al., 2017). Among viral proteins, N protein has the highest accumulation level. Accumulation of N protein accounts for a significant proportion of viral infection. We found that the nucleocapsid protein of TSWV induced autophagy. Therefore, autophagy targeting the viral N protein, which is essential for viral replication, is a “smart” strategy for a host plant to defend itself against viral infection. However, this defence is counteracted by the NSs protein encoded by TSWV. TSWV without NSs induced strong autophagy, and only in the presence of NSs was the autophagy induction suppressed. We found that NSs suppressed N‐induced autophagy. These results suggest that autophagy plays a basal defence role against TSWV, while during the co‐evolution between host autophagy and tospoviruses, TSWV has evolved the effector NSs to disable the autophagy‐dependent defence pathway.

We also found that the counter‐autophagy defence by NSs was dose‐dependent. As the concentration of NSs increases, the efficiency of inhibition on autophagy increases. However, as the amount of N protein increases, NSs cannot completely inhibit N‐induced autophagy. Although a large number of ATGs are up‐regulated in *N. benthamiana* during viral infection, autophagosome formation is only observed in the late stage of infection. This coincides with a significant increase in N protein levels during late infection stage. These results suggest that during the early infection stage, the accumulation of RNPs promotes viral replication and spread, and these RNPs may induce autophagosome formation. However, the induction of autophagy is inhibited by the viral NSs protein, and this suppression of autophagy by NSs facilitates viral replication. As the virus replicates more, the N protein is produced significantly, which further induces autophagy. At some point, however, NSs cannot completely suppress the autophagy induced by the large amounts of N protein. We speculate that this may be a result of co‐evolution between plant and TSWV. During the early stages of infection, NSs is needed to inhibit autophagy, thus favouring the virus to establish primary infection. When the virus spreads systemically, the plant needs to improve its adaptation to the virus. Thus, the interaction between virus and plant prolongs the opportunity for virus transmission by the thrips vector.

Effectors of several viruses have also been found to suppress autophagy‐mediated antiviral defence. BSMV γb protein inhibits autophagy‐mediated antiviral defence by interfering with the ATG7–ATG8 interaction to promote virus infection (Yang et al., 2018). Geminiviral (TLCYnV) C2 protein inhibits autophagy by impairing the interaction of ATG7 and ATG8 (Cao et al., 2023). CLCuMuB βC1 disrupts autophagy by interfering with the interactions between ATG3 and GAPCs (Ismayil et al., 2020). Conversely, CWMV CP promotes the interaction between GAPC2 and ATG3 to suppress autophagy (Ismayil et al., 2020; Niu et al., 2022). We tried to investigate the molecular mechanism underlying how NSs suppresses autophagy. Unfortunately, we did not observe any effect of NSs on either ATG7–ATG8 or ATG3–GAPC interactions. NSs also did not affect the interactions between other ATGs either. ATG6 is a component of the phosphatidylinositol 3‐kinase (PtdIns3K) complex and positively regulates vesicle nucleation during autophagosome formation (Funderburk et al., 2010). We found that NSs interacted with ATG6 in planta and inhibited ATG6‐induced autophagy. This finding suggests that NSs interferes with autophagy induction mediated by ATG6. The mechanism by which NSs suppresses autophagy through the ATG6 pathway deserves further investigation in the future.

In conclusion, not only RNAi and ETI play important roles in antiviral defence against TSWV, a segmented negative‐strand RNA virus, but autophagy also contributes to antiviral defence against TSWV. The N protein encoded by TSWV induces autophagy. However, this autophagy‐mediated defence is counteracted by the viral effector NSs. These findings provide new insights into antiviral defence and counterdefence mechanism for host plant and negative‐strand RNA viruses.

## 4 EXPERIMENTAL PROCEDURES

### 4.1 Plant materials and growth conditions

*Nicotiana benthamiana* and *A. thaliana* seedlings were grown in an insect‐free growth chamber at 23°C and 16 h light/8 h dark photoperiod. The *atg5* mutant *Arabidopsis* seeds and GFP‐AtATG8e transgenic *Arabidopsis* seeds were kindly provided by Professor Richard D. Vierstra Washington University and Professor Shi Xiao at Zhongshan University, respectively.

### 4.2 Plasmid constructs

The constructs expressing full‐length TSWV RdRp, Gn, Gc, N, NSm and NSs were described previously (Feng et al., 2020). For the transient expression analysis, the full‐length *NSs*, *AtATG6* and *NbATG8f* genes were cloned into the binary vectors generate p2300‐mCherry‐NbATG8f and p2300‐AtATG6‐YFP. For the BiFC assay the full‐length *AtATG1*, *AtATG3*, *AtATG4*, *AtATG5*, *AtATG6*, *AtATG7*, *AtATG8a*, *AtATG8b*, *AtATG8c*, *AtATG8d*, *AtATG8e*, *AtATG8f*, *AtATG8g*, *AtATG8i*, *AtATG12a*, *AtATG12b*, *AtATG13*, *AtATG18a*, *AtATG18b*, *AtATG18c*, *AtATG18d*, *AtATG18g*, *AtGAPC1*, *AtGAPC2* and *AtVPS34* genes were cloned into BiFC vectors pCV‐nYFP and pCV‐cYFP. To silence *NbATG5* and *NbATG7*, partial cDNA fragments of these genes were amplified by RT‐PCR and then cloned into pTRV2 vector to generate pTRV2‐NbATG5 and pTRV2‐NbATG7. GenBank accession numbers of ATGs in this study are provided in Table S1. All primers used in this paper are listed in Table S2.

### 4.3 Virus inoculation

The TSWV isolate from lettuce (TSWV‐LE) (Feng et al., 2020) was used in this study. For virus inoculation, about 0.5 g fresh TSWV‐infected leaves of *N. benthamiana* was ground in 5 mL 10 mM sodium phosphate (pH 7.0) and this crude extract was mechanically inoculated onto leaves of *A. thaliana* ecotype Columbia (Col‐0) and *N. benthamiana*.

### 4.4 RNA isolation and RT‐qPCR analysis

Total RNA was extracted from collected leaf samples using the Total RNA Kit (Tiangen) as instructed. cDNA was synthesized from total RNA samples using the HiScript III RT SuperMix kit (Vazyme) followed by qPCR amplification using the AceQ qPCR SYBR Green Master Mix kit (Vazyme). *NbActin* and *AtActin* were used as internal controls for *N. benthamiana* and *Arabidopsis*, respectively. The primers used in this study is provided in Table S1. RT‐qPCR was conducted and analysed as described previously (Hong et al., 2021).

### 4.5 Transient expression

For protein transient expression analysis in *N. benthamiana* leaves, the binary vector constructs were transformed into *Agrobacterium tumefaciens* GV3101. *A. tumefaciens* transformed with plasmids was grown to OD_600_ 1.0 and resuspended in infiltration buffer (10 mM MgCl_2_, 10 mM MES, 100 μM acetosyringone). After 3 h incubation in the dark, the mixed *Agrobacterium* cultures were infiltrated into leaves of *N*. *benthamiana* plants.

### 4.6 Chemical treatments and confocal microscopy

To examine the autophagosome, the leaves were agroinfiltrated with autophagy marker mCherry‐NbATG8f for 60 h expression, followed by an additional infiltration with 50 μM E‐64d for 12 h before confocal analysis. At 2–11 days after infiltration, 1–2 cm^2^ leaf explants were excised for examination by confocal microscopy (710; Zeiss). Excitation/detection parameters for GFP and mCherry were 488 nm/490–552 nm and 561 nm/569–652 nm, respectively.

### 4.7 Immunoblotting and immunoprecipitation

Protein extractions and immunoblots were referenced from previously described (Ma et al., 2017). Total protein was extracted from agroinfiltrated leaves of *N. benthamiana*. About 1 g leaf tissue was homogenized in 2 mL extraction buffer (10 mM sodium phosphate, pH 7.0) containing protease inhibitor cocktail (Sigma). Protein samples were separated on SDS–PAGE gels, transferred to PVDF membranes, blocked with 5% skim milk solution, and incubated with anti‐FLAG (1:5000, Sigma‐Aldrich) or anti‐GFP (1:5000, Sigma‐Aldrich), or TSWV N, NSm and NSs (produced in this laboratory) antibodies at room temperature for 2 h or overnight at 4°C. After incubation in a secondary horseradish peroxidase‐conjugated goat anti‐rabbit [1:10,000, Sigma‐Aldrich] antibody for 1 h, antibodies on the blots were detected using the ECL Substrate Kit (Thermo Scientific). ECL signals were visualized using the ChemiDoc Touch Imaging System (Bio‐Rad). Blots were also stained with Ponceau S and used as reference for sample loading and quantification.

### 4.8 dsRNA silencing and virus‐induced gene silencing

To knock down *NbATG5* expression in *N. benthamiana* leaves, 300‐bp fragments, representing a partial forward or reverse sequence of *NbATG5*, were amplified and sequentially inserted into the pCambia1300S‐intron vector to generate p1300S‐intron‐NbATG5. This vector is capable of expressing an inverted repeat of *NbATG5* in plant cells. *Agrobacterium* cultures harbouring pTRV1 and TRV2‐YFP, TRV2‐NbATG5, TRV2‐NbATG7 or TRV2‐NbNBR1 were resuspended and mixed at a 1:1 ratio. After 3 h incubation, the mixed *Agrobacterium* cultures were infiltrated into leaves of 6‐week‐old *N. benthamiana* plants. TRV‐treated plants were grown in a growth chamber at 23°C and 16 h light/8 h dark photoperiod. The RNA expression level of targeted genes was analysed by RT‐qPCR. The effectiveness of the virus‐induced gene silencing assay was evaluated using the phytoene desaturase (*PDS*) gene as described previously (Ma et al., 2015). The PDS silencing phenotype typically appears in the upper leaves at 7 dpi.

## CONFLICT OF INTEREST STATEMENT

The authors declare that no competing interests exist.

## Supporting information


Figure S1.

Figure S2.

Figure S3.

Figure S4.

Figure S5.

Figure S6.

Figure S7.

Table S1.

Table S2.

## Data Availability

The data that support the findings of this study are available from the corresponding author upon reasonable request.
