# Supplementary figures and images for: Autophagy plays an antiviral defence role against tomato spotted wilt orthotospovirus and is counteracted by viral effector NSs
Source: Mol Plant Pathol. 2024 Sep 30;25(10):e70012. doi: 10.1111/mpp.70012 (PMC11442783; doi:10.1111/mpp.70012)

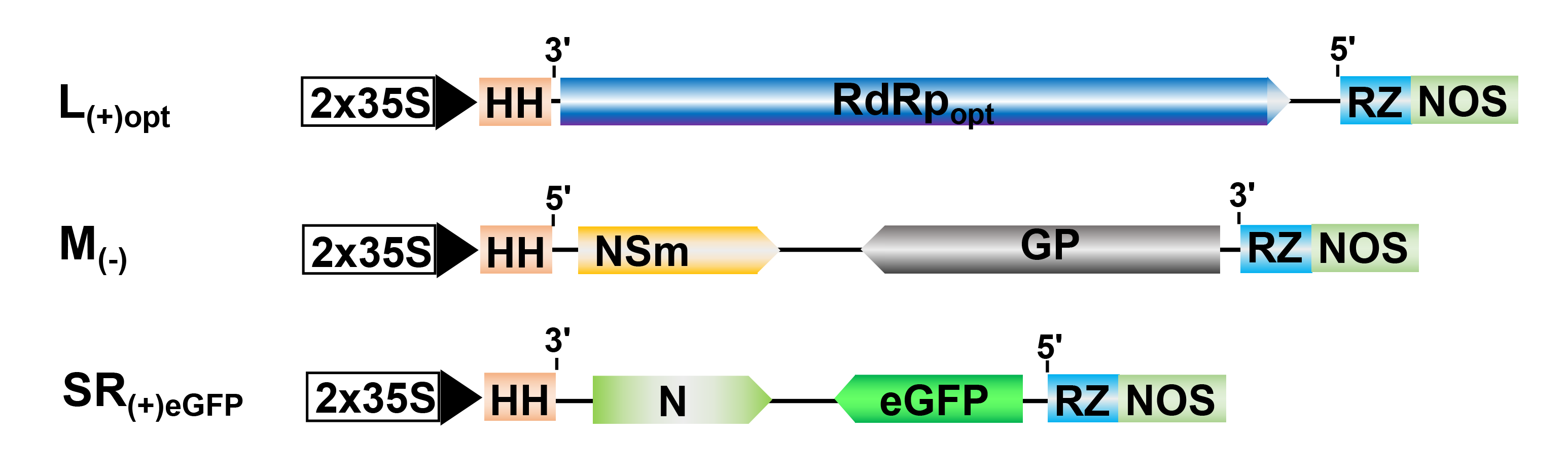

Supplement: Supplementary file 1 — Figure S1. [file MPP-25-e70012-s009.tif]

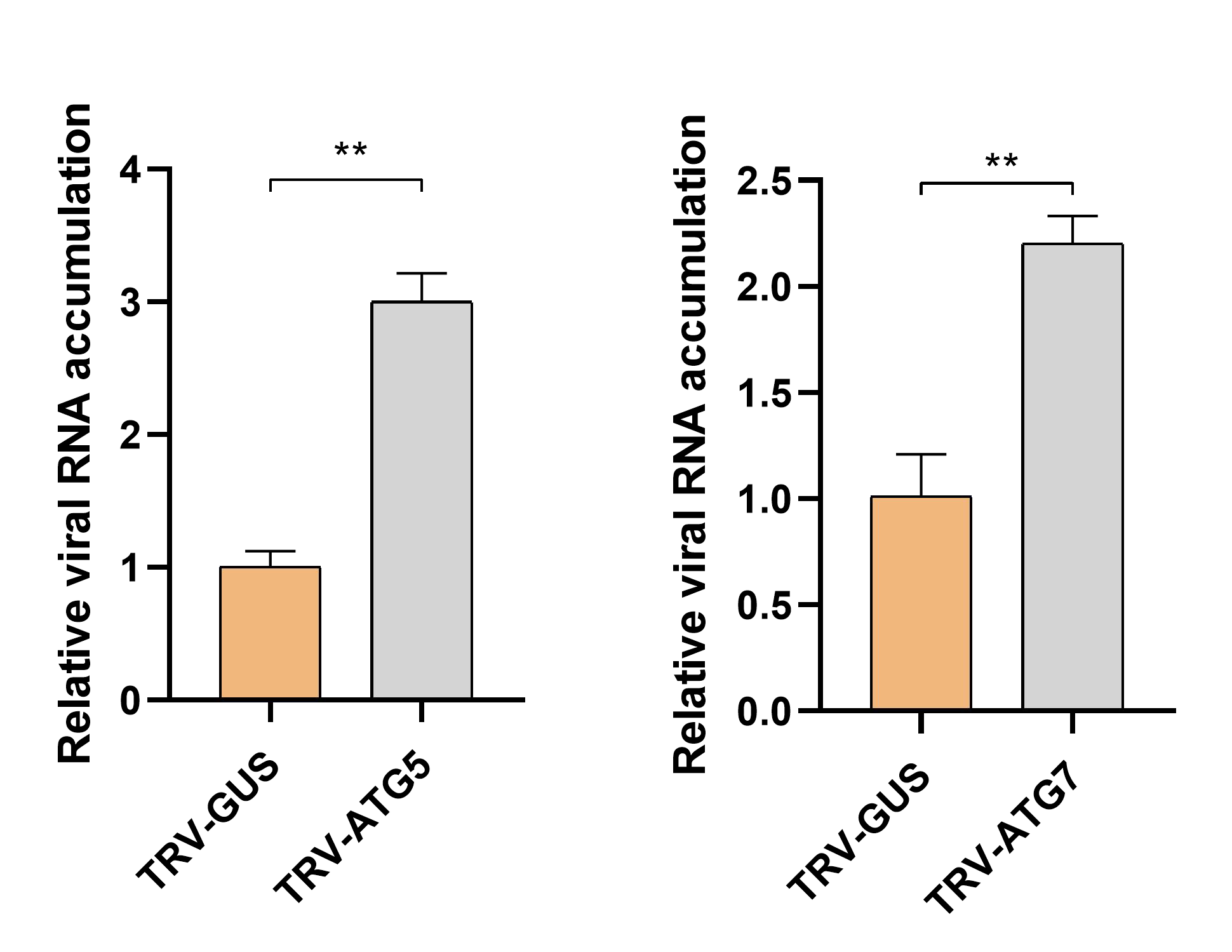

Supplement: Supplementary file 2 — Figure S2. [file MPP-25-e70012-s007.tif]

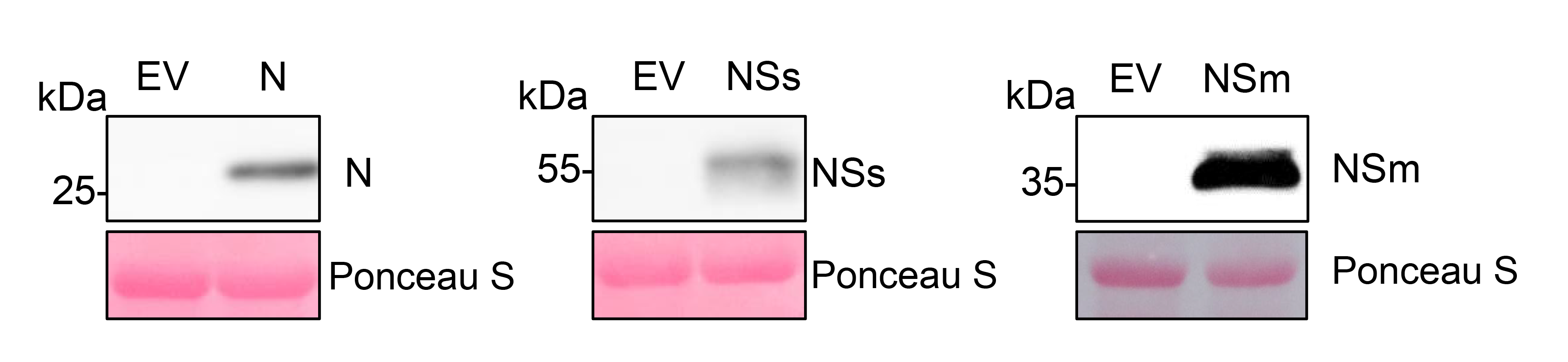

Supplement: Supplementary file 3 — Figure S3. [file MPP-25-e70012-s004.tif]

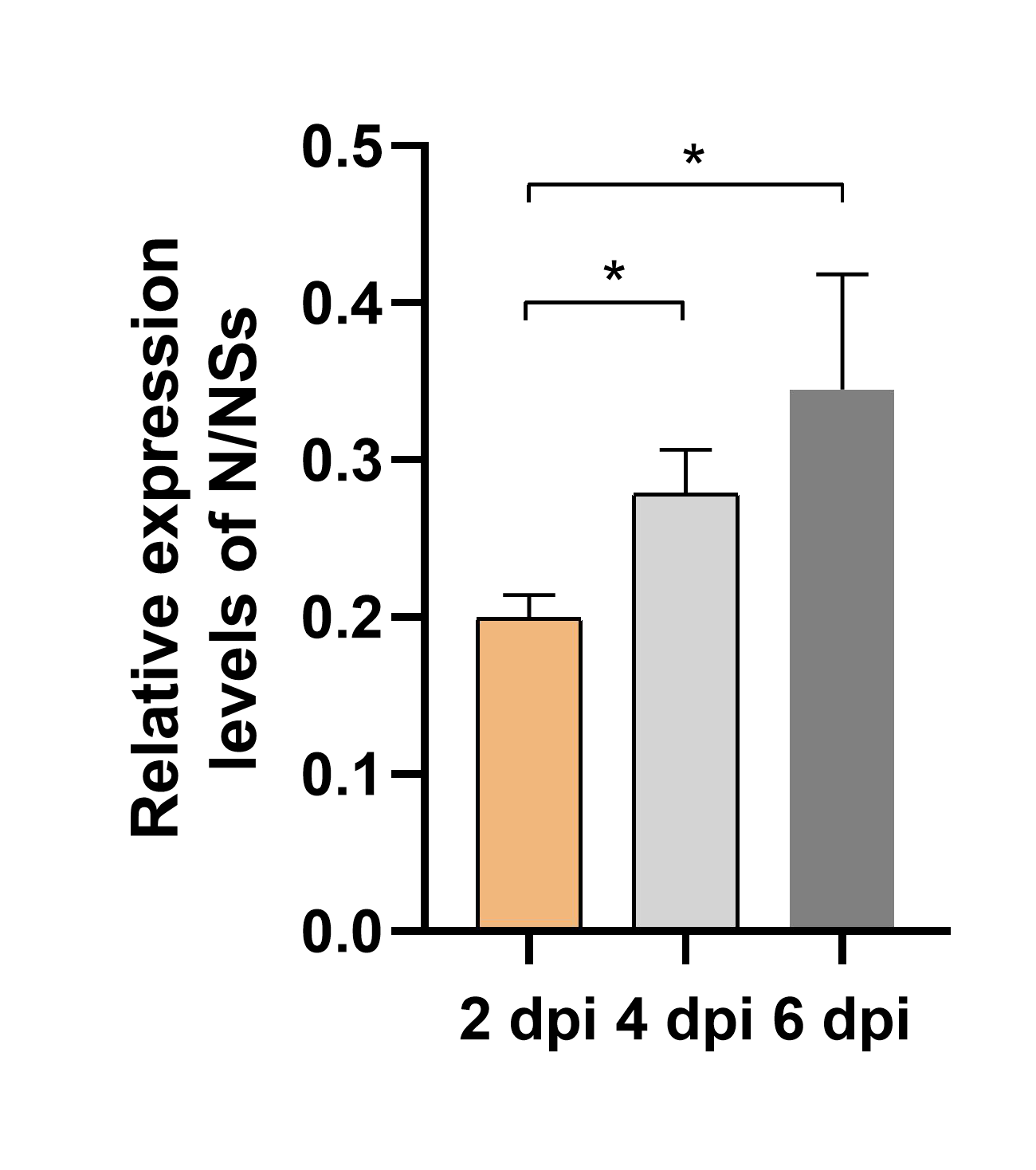

Supplement: Supplementary file 4 — Figure S4. [file MPP-25-e70012-s006.tif]

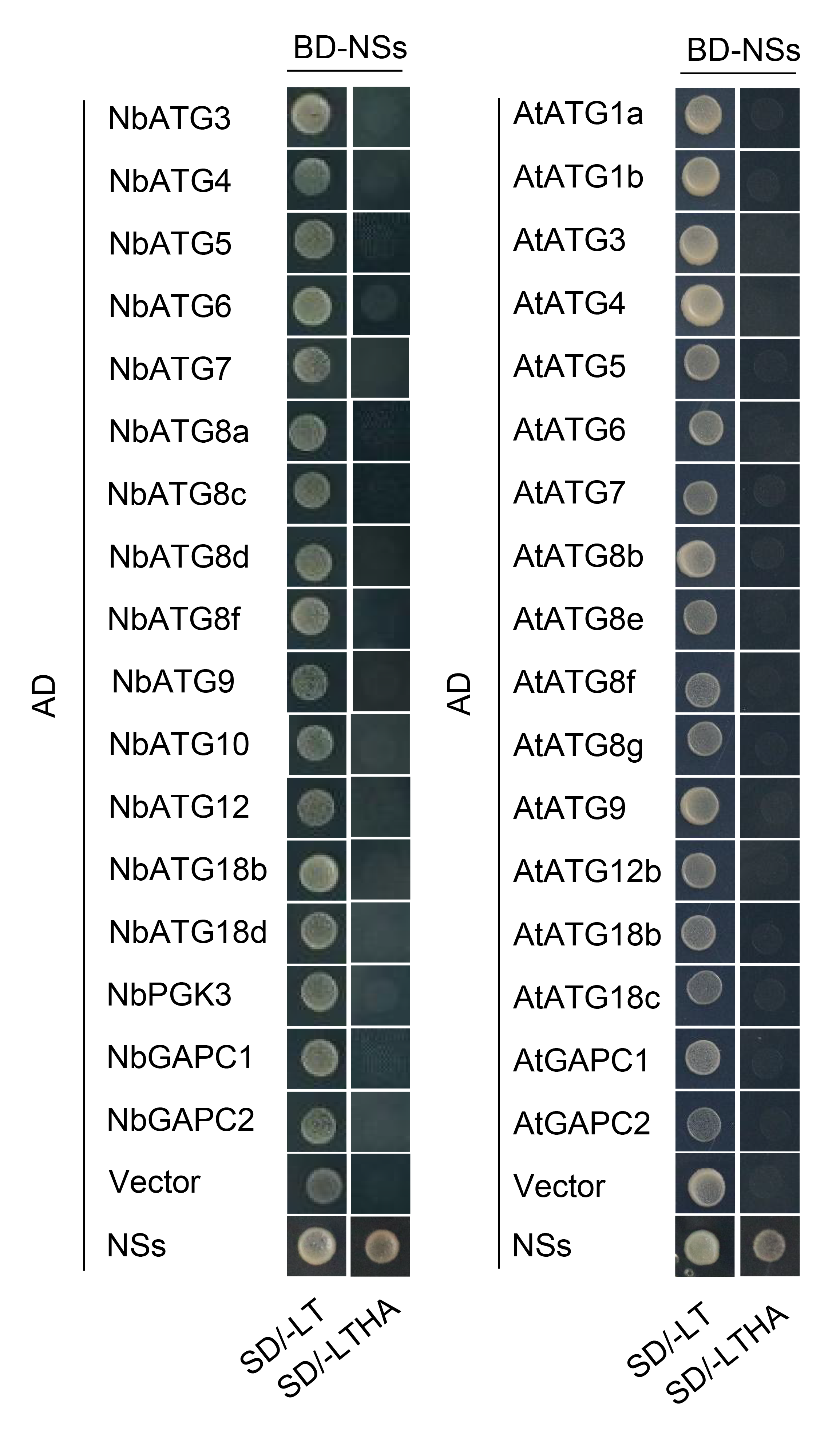

Supplement: Supplementary file 5 — Figure S5. [file MPP-25-e70012-s001.tif]

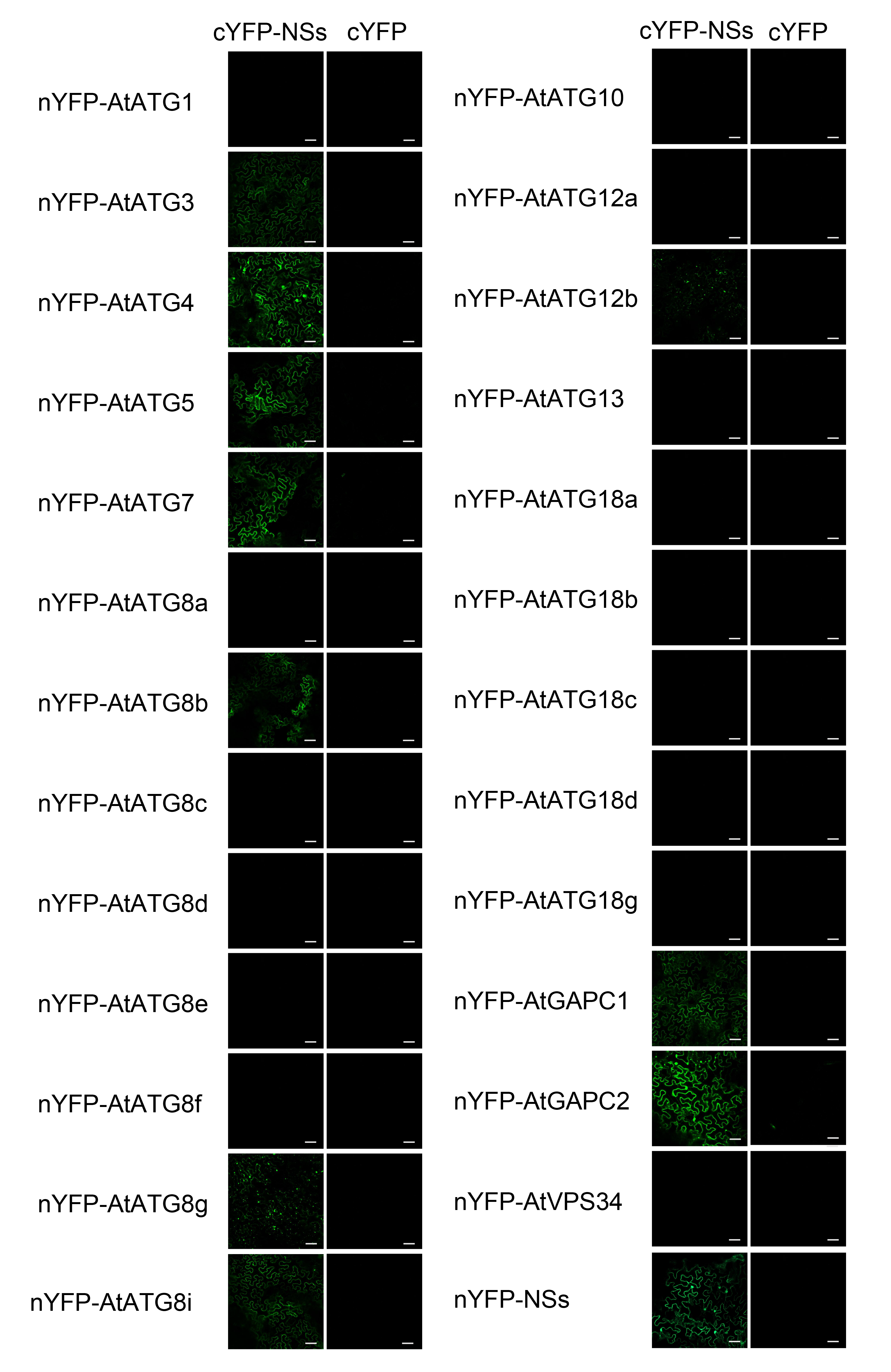

Supplement: Supplementary file 6 — Figure S6. [file MPP-25-e70012-s003.tif]

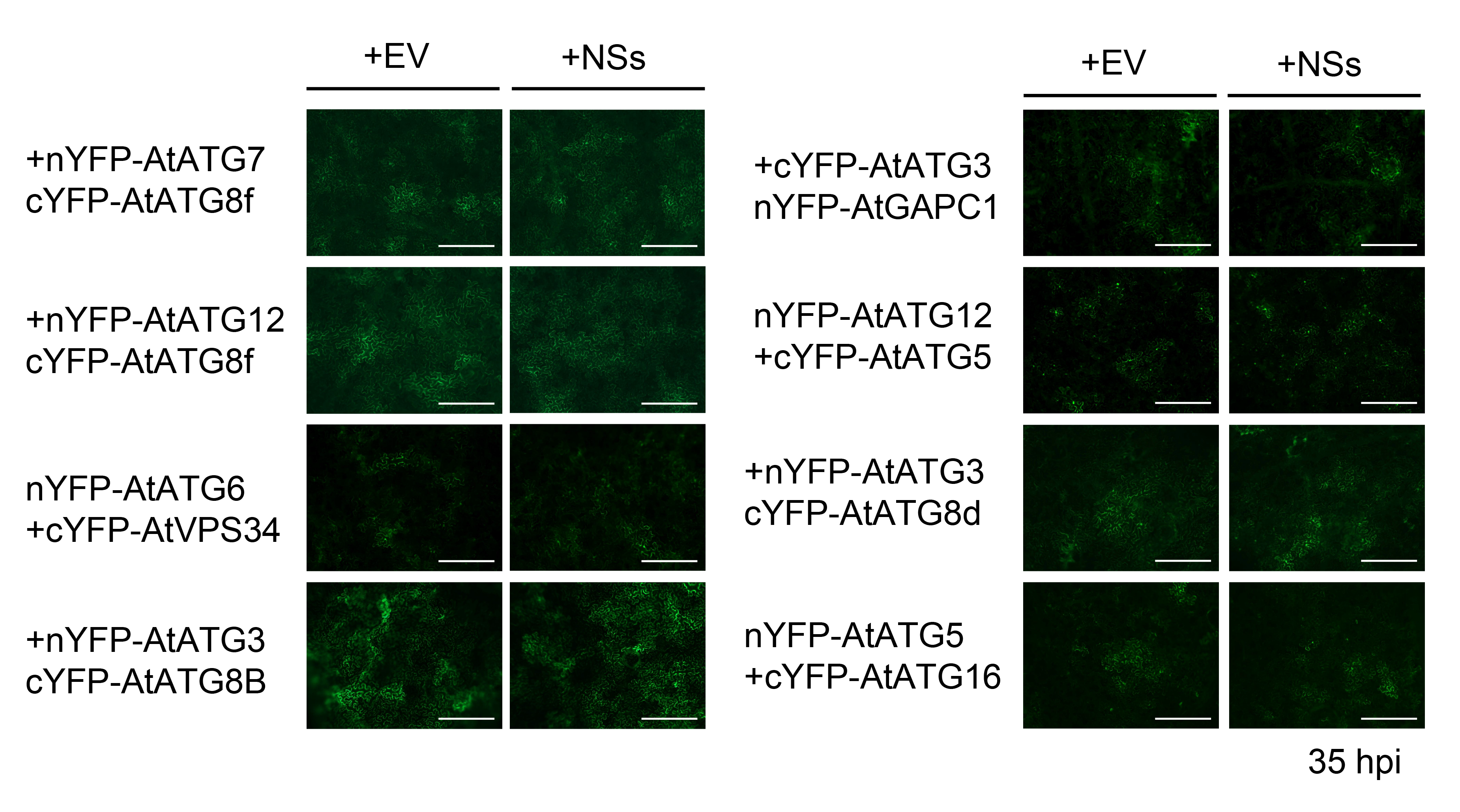

Supplement: Supplementary file 7 — Figure S7. [file MPP-25-e70012-s005.tif]
