# Supplementary material for: Autophagy plays an antiviral defence role against tomato spotted wilt orthotospovirus and is counteracted by viral effector NSs
Source: Mol Plant Pathol. 2024 Sep 30;25(10):e70012. doi: 10.1111/mpp.70012 (PMC11442783; doi:10.1111/mpp.70012)
Supplement: Supplementary file 8 — Table S1. [file MPP-25-e70012-s002.docx]

Table S1. Autophagy protein genes used in the yeast two-hybrids and BiFC.

| Gene ID | Gene name |
| --- | --- |
| AT3g61960 | AtATG1a |
| AT3g53930 | AtATG1b |
| AT5g61500 | AtATG3 |
| AB073172 | AtATG4 |
| AT5g17290 | AtATG5 |
| AT3g61710 | AtATG6 |
| AT5g45900 | AtATG7 |
| AT4g21980 | AtATG8a |
| AT4g04620 | AtATG8b |
| AT1g62040 | AtATG8c |
| AT2g05630 | AtATG8d |
| AT2G45170 | AtATG8e |
| AT4g16520 | AtATG8f |
| AT3g60640 | AtATG8g |
| AT3g06420 | AtATG8h |
| AT3g15580 | AtATG8i |
| AT2g31260 | AtATG9 |
| AT3G07525 | AtATG10 |
| AT1g54210 | AtATG12a |
| AT3g13970 | AtATG12b |
| AT3G49590 | AtATG13 |
| AT3G62770 | AtATG18a |
| AT4g30510 | AtATG18b |
| AT2G40810 | AtATG18c |
| AT3G56440 | AtATG18d |
| AT1G03380 | AtATG18g |
| AT1G60490 | AtVPS34 |
| KX369396.1 | NbATG3 |
| XM016587724 | NbATG4 |
| KX369397.1 | NbATG5 |
| AY701316 | NbATG6 |
| KX369398 | NbATG7 |
| KX120976 | NbATG8a |
| MG733101 | NbATG8c |
| KX369400 | NbATG8d |
| KU561372 | NbATG8f |
| KX369399 | NbATG9 |
| Niben101Scf01734g00001.1 | NbATG10 |
| XM016602097 | NbATG12 |
| XM019390511 | NbATG18d |
| XM019400561 | NbATG18b |
| XM016644820 | NbPGK |
| KM986323 | NbGAPC1 |
| KM986325 | NbGAPC2 |

Sequence data described in this article can be found at TAIR (https://www.arabidopsis.org) and Sol Genomics Network (https://solgenomics.net).
