# Supplementary material for: Autophagy plays an antiviral defence role against tomato spotted wilt orthotospovirus and is counteracted by viral effector NSs
Source: Mol Plant Pathol. 2024 Sep 30;25(10):e70012. doi: 10.1111/mpp.70012 (PMC11442783; doi:10.1111/mpp.70012)
Supplement: Supplementary file 9 — Table S2. [file MPP-25-e70012-s008.docx]

**Table S2**. Primers used in this study.

| **Prime name** | **Primers Sequence (5'-3')** |
| --- | --- |
| pTRV2-NbATG5 | F: CGGGATCCAATCCGAAGTCACTACTCTCCC |
|  | R: GGGGTACCGGCGAAGATAGTGCAAAGTGGA |
| pTRV2-NbATG7 | F: CGGGATCCTGGCGGATAGTGGAAGAGG |
|  | R: GGGGTACCCAACGTGTTTGTATTGAGAAG |
| qRT-PCR-NbActin | F: GGCATTCATGAAACCACATACA |
|  | R: AGGACAATGTTTCCGTACAGAT |
| qRT-PCR-NbATG5 | F: TCCCAACCTGACCAATTAGA |
|  | R: CCTAACATACAGTCGAACCG |
| qRT-PCR-NbATG7 | F: ATGGCGGATAGTGGAAGAGG |
|  | R: GAGGCTCTGCTTATCAAGCG |
| qRT-PCR-AtActin | F: GAGTCGACGCACAGGGTACC |
|  | R: CCGTCGGGTAATTCATAGTTCTT |
| qRT-PCR-AtATG5 | F: ATGGCGAAGGAAGCGGTCAA |
|  | R: TCGGGTTCTGCACAAAGGAG |
| qRT-PCR-AtATG7 | F: ATGGCTGAGAAAGAAACTCC |
|  | R: CCCAGGAACAGGACACTTAT |
| qRT-PCR-TSWV N | F: TTGACACAAGGCAAAGATCT |
|  | R: CCCTGATCAAGCTATCAAGC |
| qRT-PCR-TSWV NSs | F: TGTCCATGAATGGCTGTACT |
|  | R: CTCCTGCAACTTTGAACAAT |
| p1300S- RNAi-dsNbATG5 | F: TTTGCAGGTATTTCTAGATTTAGCTGGTCCTGTGCCAT |
|  | R: GCATGCCTGCAGGTCGACCCTGGAAATATTTTAACACC |
| p2300-AtATG6-YFP | F: TTTCGCGAGCTCGGTACCATGAGGAAAGAGGAGATTCC |
|  | R: CACCATGGATCCTCTAGACTAAGTTTTTTTACATGAAG |
| p2300-mCherry-NbATG8f | F: TACAGATCTGGATCCATGGCTAAGAGCTCATTCAA |
|  | R: CAGGTCGACTCTAGACTACAGCTTGTTCAGGTCCC |
| pGADT7-NbATG3 | F: CATGGAGGCCAGTGAATTCATGGTACTGTCGCAGAAGAT |
|  | GCTCGAGCTCGATGGATCCTCAGGTGCTGCTGCTACCAA |
| pGADT7-NbATG4 | F: ATGGAGGCCAGTGAATTCATGCCTAAAAAAACCCAATT |
|  | R: CTCGAGCTCGATGGATCCTCAAAGGAGTTGCCACTCAT |
| pGADT7-NbATG5 | F: CATGGAGGCCAGTGAATTCATGGGAAGTAAAGGGGCAGG |
|  | R: CGAGCTCGATGGATCCTCACTATATGGTGATGGGTTCTT |
| pGADT7-NbATG6 | F: CATGGAGGCCAGTGAATTCATGATGAAAAATAGCAGCAG |
|  | R: CGAGCTCGATGGATCCTCATCAAGATTGAAACTTGGTAT |
| pGADT7-NbATG7 | F: GAGGCCAGTGAATTCATGGCGGATAGTGGAAGAGG |
|  | R: GAGCTCGATGGATCCTTATATTTCTATAGAGTCATC |
| pGADT7NbATG8a | F: GAGGCCAGTGAATTCATGGCCAAAAGCTCCTTCAA |
|  | R: CGAGCTCGATGGATCCTCAGAACGATCCGAATGTATTCT |
| pGADT7-NbATG8c | F: GAGGCCAGTGAATTCATGGCGAAGAGTTCTTTCAA |
|  | R: CGAGCTCGATGGATCCTCATTAATTGCCGAGCTCAAGAAA |
| pGADT7-NbATG8d | F: GAGGCCAGTGAATTCATGGCCGAAGCTGCTCGTAT |
|  | R: CGAGCTCGATGGATCCTCAAGATTTGCAGAGAGAAAGCT |
| pGADT7-NbATG8f | F: ATGGAGGCCAGTGAATTCATGGCTAAGAGCTCATTCAA |
|  | R: CTCGAGCTCGATGGATCCCTACAGCTTGTTCAGGTCCC |
| pGADT7-NbATG9 | F: ATGGAGGCCAGTGAATTCATGATGTTTGGTGGACAAAA |
|  | R: GCTCGAGCTCGATGGATCCATCTTGACTTCTATCAGAGA |
| pGADT7-NbATG10 | F: ATGGAGGCCAGTGAATTCATGATTGACATCTCCTCGTG |
|  | R: GCTCGAGCTCGATGGATCCCTATAATACATTAGAGACAT |
| pGADT7-NbATG12 | F: ATGGAGGCCAGTGAATTCATGGCCTCCGATTCTCGTAA |
|  | R: CTCGAGCTCGATGGATCCCTAACCGTTTCCTGCATTGT |
| pGADT7-NbATG18B | F: CATGGAGGCCAGTGAATTCATGGCAAATCAATCCTCTTC |
|  | R: CTCGAGCTCGATGGATCCTCATGATAATGAGGATGTTT |
| pGADT7-NbATG18D | F: ATGGAGGCCAGTGAATTCATGACTACTCTATCATCCCC |
|  | R: CTCGAGCTCGATGGATCCCTATCTCTGCCTACTCTCTG |
| pGADT7-NbGAPC1 | F: ATGGAGGCCAGTGAATTCATGGCATCTGACAAGAAGAT |
|  | R: CTCGAGCTCGATGGATCCTTATGCAACAGAAGCCATAT |
| pGADT7-NbGAPC2 | F: ATGGAGGCCAGTGAATTCATGGCCAAGGTTAAGATTGG |
|  | R: CTCGAGCTCGATGGATCCTTACTGGACTGATGCCATGT |
| pGADT7-NbPGK3 | F: TGGAGGCCAGTGAATTCATGGCAGTGAAGAAGAGTGT |
|  | R: AGCTCGATGGATCCTCATTAAGCATCATCGAGAGCAA |
| pGADT7-AtATG1a | F: GAGGCCAGTGAATTCATGGAGTCGGCACGACTTGT |
|  | R: CGAGCTCGATGGATCCTCAGCCCCACCACTCTTGTG |
| pGADT7-AtATG1b | F: GAGGCCAGTGAATTCATGGCTCGGCTTAATAAGAA |
|  | R: CGAGCTCGATGGATCCTCATTGCTTCTGAGGATATG |
| pGADT7-AtATG3 | F: GAGGCCAGTGAATTCCTACGGAGAGCTGCTTCTGT |
|  | R: CGAGCTCGATGGATCCTCAGGTGCTTGAGCTACCGA |
| pGADT7-AtATG4 | F: GAGGCCAGTGAATTCATGAAGGCTATATGTGATAG |
|  | R: CGAGCTCGATGGATCCTCAAAGTAATTGCCAGTCAT |
| pGADT7-AtATG5 | F: GAGGCCAGTGAATTCATGGCGAAGGAAGCGGTCAA |
|  | R: CGAGCTCGATGGATCCTCACCTTTGAGGAGCTTTCA |
| pGADT7-AtATG6 | F: GAGGCCAGTGAATTCATGAGGAAAGAGGAGATTCC |
|  | R: CGAGCTCGATGGATCCTCAAGTTTTTTTACATGAAG |
| pGADT7-AtATG7 | F: GAGGCCAGTGAATTCATGGCTGAGAAAGAAACTCC |
|  | R: CGAGCTCGATGGATCCTCAAAGATCTACAGCTACAT |
| pGADT7-AtATG8b | F: GAGGCCAGTGAATTCATGGAGAAGAACTCCTTCAA |
|  | R: CGAGCTCGATGGATCCTCAGCAGTAGAAAGATCCAC |
| pGADT7-AtATG8e | F: GAGGCCAGTGAATTCATGAATAAAGGAAGCATCTT |
|  | R: CGAGCTCGATGGATCCTCAGATTGAAGAAGCACCGA |
| pGADT7-AtATG8f | F: GAGGCCAGTGAATTCATGATTCATGTGGCTACGTG |
|  | R: CGAGCTCGATGGATCCTCATGGAGATCCAAATCCAA |
| pGADT7-AtATG8g | F: GAGGCCAGTGAATTCATGAGTAACGTCAGCTTCAG |
|  | R: CGAGCTCGATGGATCCTCAAGTCATTGACGATCCAA |
| pGADT7-AtATG9 | F: GAGGCCAGTGAATTCATGATGAGCAGTGGGCATAA |
|  | R: CGAGCTCGATGGATCCTCATCACCGTAATGTGGTGCTTG |
| pGADT7-AtATG12b | F: GAGGCCAGTGAATTCATGGCGACCGAATCTCCGAA |
|  | R: CGAGCTCGATGGATCCTCAGGAGCATGGTACGAATG |
| pGADT7-AtATG18b | F: GAGGCCAGTGAATTCATGTTGTATAGCTCAGATCT |
|  | R: CGAGCTCGATGGATCCTCACCCGGTGGTTATGGAGAAGA |
| pGADT7-AtATG18c | F: GAGGCCAGTGAATTCATGAGTTCAACTGTTTCAAA |
|  | R: CGAGCTCGATGGATCCTCACGGGCGGTTGTCCATCTTCA |
| pGADT7-AtGAPC1 | F: GAGGCCAGTGAATTCATGGCTGACAAGAAGATTAG |
|  | R: CGAGCTCGATGGATCCTCATTAGGCCTTTGACATGTGGA |
| pGADT7-AtGAPC2 | F: GAGGCCAGTGAATTCATGGCTGACAAGAAGATCAG |
|  | R: CGAGCTCGATGGATCCTCATTAGGCCTTTGACATGTGAA |
| pCV-nYFP-AtATG1a | F: TCGACGCACAGGGTACCATGGAGTCGGCACGACTTGT |
|  | R: AGCTCGCCTGGGGATCCGCCCCACCACTCTTGTG |
| pCV-nYFP-AtATG3 | F: TCGACGCACAGGGTACCCTACGGAGAGCTGCTTCTGT |
|  | R: AGCTCGCCTGGGGATCCGGTGCTTGAGCTACCGA |
| pCV-nYFP-AtATG4 | F: TCGACGCACAGGGTACCATGAAGGCTATATGTGATAG |
|  | R: AGCTCGCCTGGGGATCCAAGTAATTGCCAGTCAT |
| pCV-nYFP-AtATG5 | F: TCGACGCACAGGGTACCATGGCGAAGGAAGCGGTCAA |
|  | R: AGCTCGCCTGGGGATCCCCTTTGAGGAGCTTTCA |
| pCV-nYFP-AtATG6 | F: TCGACGCACAGGGTACCATGAGGAAAGAGGAGATTCC |
|  | R: AGCTCGCCTGGGGATCCAGTTTTTTTACATGAAG |
| pCV-nYFP-AtATG7 | F: TCGACGCACAGGGTACCATGGCTGAGAAAGAAACTCC |
|  | R: AGCTCGCCTGGGGATCCAAGATCTACAGCTACAT |
| pCV-nYFP-AtATG8a | F: TCGACGCACAGGGTACCATGGCTAAGAGTTCCTTCAA |
|  | R: AGCTCGCCTGGGGATCCAGCAACGGTAAGAGATC |
| pCV-nYFP-AtATG8b | F: TCGACGCACAGGGTACCATGGAGAAGAACTCCTTCAA |
|  | R: AGCTCGCCTGGGGATCCGCAGTAGAAAGATCCAC |
| pCV-nYFP-AtATG8c | F: TCGACGCACAGGGTACCATGGCTAATAGCTCTTTCAA |
|  | R: AGCTCGCCTGGGGATCCAACCAAACCAAAGGTGT |
| pCV-nYFP-AtATG8d | F: TCGACGCACAGGGTACCATGGCGATTAGCTCCTTCAA |
|  | R: AGCTCGCCTGGGGATCCGAAGAAGATCCCGAACG |
| pCV-nYFP-AtATG8e | F: TCGACGCACAGGGTACCATGAATAAAGGAAGCATCTT |
|  | R: AGCTCGCCTGGGGATCCGATTGAAGAAGCACCGA |
| pCV-nYFP-AtATG8f | F: TCGACGCACAGGGTACCATGATTCATGTGGCTACGTG |
|  | R: AGCTCGCCTGGGGATCCTGGAGATCCAAATCCAA |
| pCV-nYFP-AtATG8g | F: TCGACGCACAGGGTACCATGAGTAACGTCAGCTTCAG |
|  | R: AGCTCGCCTGGGGATCCAGTCATTGACGATCCAA |
| pCV-nYFP-AtATG8i | F: TCGACGCACAGGGTACCATGAAATCGTTCAAGGAACA |
|  | R: AGCTCGCCTGGGGATCCTCAACCAAAGGTTTTCTCAC |
| pCV-nYFP-AtATG10 | F: TCGACGCACAGGGTACC ATGGATTCAGCTCGAGAGGT |
|  | R: AGCTCGCCTGGGGATCCATTCAGCATCTCAAGAG |
| pCV-nYFP-tATG12a | F: TCGACGCACAGGGTACCATGGCGACGGAGTCGTCGTC |
|  | R: AGCTCGCCTGGGGATCCGCCCCATGCCATGGAAC |
| pCV-nYFP-tATG12b | F: TCGACGCACAGGGTACCATGGCGACCGAATCTCCGAA |
|  | R: AGCTCGCCTGGGGATCCGGAGCATGGTACGAATG |
| pCV-nYFP-AtATG13 | F: TCGACGCACAGGGTACCATGGATTTTCCAGAGAATTT |
|  | R: AGCTCGCCTGGGGATCCGTGGACGCGAGTTGGTC |
| pCV-nYFP-tATG18a | F: TCGACGCACAGGGTACCATGGCCACCGTATCTTCTTC |
|  | R: AGCTCGCCTGGGGATCCCCTCCCATCCATGCCAAGAA |
| pCV-nYFP-tATG18b | F: TCGACGCACAGGGTACCATGTTGTATAGCTCAGATCT |
|  | R: AGCTCGCCTGGGGATCCCCCGGTGGTTATGGAGAAGA |
| pCV-nYFP-tATG18c | F: TCGACGCACAGGGTACCATGAGTTCAACTGTTTCAAA |
|  | R: AGCTCGCCTGGGGATCCCGGGCGGTTGTCCATCTTCA |
| pCV-nYFP-tATG18d | F: TCGACGCACAGGGTACCATGGATCCTCGGAGAAACTT |
|  | R: AGCTCGCCTGGGGATCCTTTTTTCAGATATCTCCTAA |
| pCV-nYFP-tATG18g | F: TCGACGCACAGGGTACCATGATGAAGAAGGGGAAAGG |
|  | R: AGCTCGCCTGGGGATCCATCACCTACAAAGGAAACCA |
| pCV-nYFP-tGAPC1 | F: TCGACGCACAGGGTACCATGGCTGACAAGAAGATTAG |
|  | R: AGCTCGCCTGGGGATCCTTAGGCCTTTGACATGTGGA |
| pCV-nYFP-tGAPC2 | F: TCGACGCACAGGGTACCATGGCTGACAAGAAGATCAG |
|  | R: AGCTCGCCTGGGGATCCTTAGGCCTTTGACATGTGAA |
